# Disulfidptosis‐ and Ferroptosis‐Related Gene Signatures in Rheumatoid Arthritis: Association Analysis and Diagnostic Model Construction

**DOI:** 10.1002/kjm2.70287

**Published:** 2026-09-03

**Authors:** Ting‐Ting Wang, Dong‐Mei Wang, Xue Li, Yuan‐Li Wei, Ping‐Dan Liu, Shi‐Lin Li, Si‐Ying Xiong, Cai‐Zhen Liu, Fan‐Xin Zeng, Jian‐Hong Wu, Lin Tang

**Affiliations:** ^1^ Department of Rheumatology and Immunology The Second Affiliated Hospital of Chongqing Medical University Chongqing China; ^2^ Department of Rheumatology and Immunology Dazhou Central Hospital Dazhou China; ^3^ Department of Clinical Research Center Dazhou Central Hospital Dazhou China

**Keywords:** diagnostic model, disulfidptosis, ferroptosis, rheumatoid arthritis, single‐cell Mendelian randomization

## Abstract

Rheumatoid arthritis (RA) is a chronic autoimmune disease characterized by persistent synovial inflammation and progressive joint damage. Although ferroptosis has been implicated in RA progression, the role of disulfidptosis and its interaction with ferroptosis remains unclear. This study aimed to identify Disulfidptosis‐ and Ferroptosis‐Related Genes (DFRGs) associated with RA and construct a candidate diagnostic model. Blood transcriptomic data from 154 RA patients and 43 healthy controls were analyzed. Differentially expressed DFRGs were identified, and immune infiltration was assessed using single‐sample Gene Set Enrichment Analysis (ssGSEA). Feature genes were selected through correlation analysis and logistic regression, followed by construction of a diagnostic nomogram. External validation was performed using independent datasets. Potential regulatory miRNAs and therapeutic agents were predicted, and single‐cell eQTL‐based Mendelian randomization (MR) was used to evaluate genetic associations between core gene expression and RA risk. Eleven DFRGs were identified and were enriched in p53, mTOR, and immune‐related pathways. Five feature genes, DUOX1, DRD4, ATP6V1G2, ASNS, and CDKN1A, were used to establish a diagnostic model with favorable performance in the training cohort and external validation datasets. Five key miRNAs and 61 potential therapeutic agents were predicted. MR suggested that ASNS expression may be protective, whereas ATP6V1G2 expression may increase RA risk. These findings suggest that DFRGs‐related signatures may be involved in RA pathogenesis and immune dysregulation, although further in vitro and in vivo validation is required.

## 1 Introduction

Rheumatoid arthritis (RA) is an autoimmune and inflammatory disease that causes joint destruction. It is characterized by symmetrical polyarticular synovitis, cartilage damage, bone erosion, and joint deformity, ultimately leading to persistent disability, joint damage, and shortened life expectancy. RA affects approximately 0.5%–1% of the global population [1, 2, 3]. In recent years, significant advancements in RA treatment have been made, including the development of biological agents and small molecule targeted drugs. However, current diagnostic and therapeutic approaches still face limitations in improving accuracy, personalization, and mitigating adverse effects. Notably, emerging forms of programmed cell death, particularly ferroptosis and the newly discovered disulfidptosis, may reveal unexplored pathogenic mechanisms in RA. By integrating dual death pathways for the first time, this study aims to uncover synergistic targets for precise diagnosis and therapy.

Ferroptosis is a distinctive form of regulated cell death resulting from iron accumulation, excessive reactive oxygen species (ROS) production, and heightened lipid peroxidation. Recent studies have highlighted the involvement of ferroptosis in the pathogenesis of various autoimmune diseases, including RA [4]. Evidence suggests that ferroptosis occurs in fibroblast‐like synoviocytes (FLSs), macrophages, and chondrocytes within the RA joint, contributing to inflammation and tissue destruction [5]. Furthermore, key regulators of ferroptosis such as GPX4 and SLC7A11 are reduced in RA synovial cells, suggesting that targeting ferroptosis could represent a promising therapeutic strategy [6]. Disulfidptosis has been identified as a novel form of regulated cell death triggered by aberrant disulfide bond accumulation within the actin cytoskeleton [7]. Given the established links between cytoskeletal dynamics, immune cell function, and autoimmunity [8, 9], disulfidptosis may be hypothesized to be relevant to RA pathophysiology [10]; however, its definition in this study is derived from a gene signature‐based hypothesis rather than from direct biological experimental evidence.

We hypothesized that genes associated with both ferroptosis and disulfidptosis (hereafter referred to as disulfidptosis‐ferroptosis related genes, DFRGs) might play coordinated roles in RA. This study aims to systematically identify potential key genes and regulatory networks related to disulfidptosis using bioinformatics methods and public databases, providing a theoretical basis and data foundation for subsequent experimental validation. To investigate this, we analyzed the expression of a set of DFRGs, defined as genes known to be involved in either ferroptosis or disulfidptosis pathways based on existing literature, in RA patients compared to healthy controls using transcriptome data. By constructing a diagnostic model based on key DFRGs and evaluating the immune microenvironment, we aimed to gain new insights into RA's molecular mechanisms and explore potential biomarkers for improved diagnosis. Additionally, we investigated potential upstream miRNAs and candidate drugs targeting these pathways.

## 2 Materials and Methods

**FIGURE 1:**
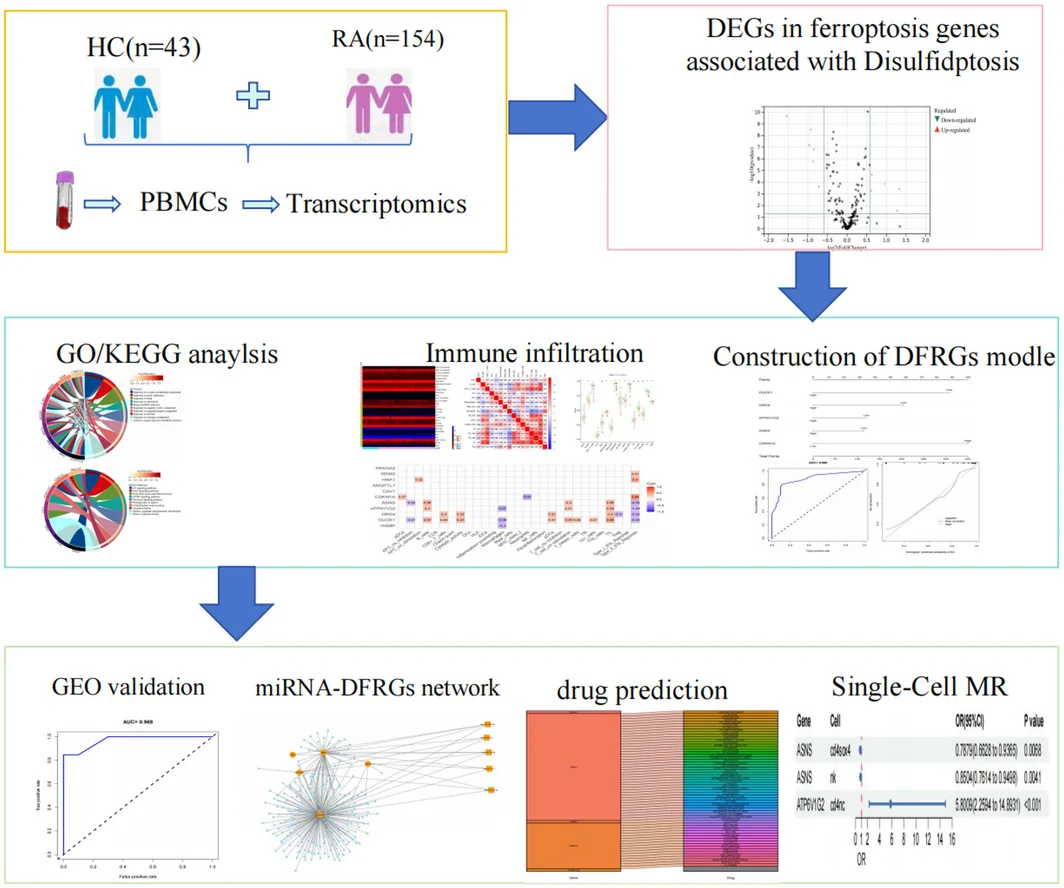
Flowchart of the study design. DFRGs, disulfidptosis‐ and ferroptosis‐related genes; HC, healthy controls; PBMCs, peripheral blood mononuclear cells; RA, rheumatoid arthritis.

The workflow of this study was illustrated in Figure 1.

### 2.1 RNA Sequencing

#### 2.1.1 Case Collection and Grouping

We selected a total of 154 patients with RA who were admitted to the Department of Rheumatology and Immunology at Dazhou Central Hospital between 1 July 2021 and 31 December 2022 as the RA group. The healthy control group consisted of 43 individuals who underwent routine physical examinations at the hospital. This study was approved by the Institutional Ethics Committee of Dazhou Central Hospital (approval number: 2021‐022) and was conducted in accordance with the Declaration of Helsinki. All participants voluntarily took part in the study and provided informed consent.

##### 2.1.1.1 Inclusion Criteria

Patients who met the diagnostic criteria for rheumatoid arthritis according to either the 1987 American College of Rheumatology or the 2010 American College of Rheumatology/European League Against Rheumatism guidelines were included. The age of the participants was ≥ 18 years, irrespective of gender.

##### 2.1.1.2 Exclusion Criteria

Patients with acute or chronic infectious diseases, significant medical conditions affecting the heart, brain, liver, kidney, or blood, malignant tumors, or mental disorders, and pregnant or lactating women were excluded.

Of the participants, 10 met the 1987 criteria, while 144 satisfied the 2010 criteria. A detailed list of clinical data for each subject is provided in Table S1, including gender, age, number of joints with tenderness, number of joints with swelling, C‐reactive protein (CRP, mg/L), rheumatoid factor (RF, IU/mL), erythrocyte sedimentation rate (ESR, mm/h), anti‐cyclic citrullinated peptide (Anti‐CCP, RU/mL), and the 28‐joint disease activity score (DAS28). The HC group consisted of 43 individuals (32 females and 11 males), while the RA group included 154 individuals (125 females and 29 males). No significant difference in sex distribution was observed between the two groups (*p* = 0.32). However, the RA group was significantly older than the HC group (55.02 ± 11.27 years vs. 49.77 ± 4.57 years, *p* < 0.0001), indicating that age may represent a potential confounding factor in subsequent analyses.

#### 2.1.2 Sample Collection and Preparation

After enrollment, a fasting venous blood sample (2.0–3.0 mL) was collected from both the RA group and the control group. Subsequently, peripheral blood mononuclear cells (PBMCs) were isolated using Ficoll‐Paque PLUS density gradient centrifugation (GE Healthcare) at 400 × g for 30 min at room temperature, followed by two washes in phosphate‐buffered saline (PBS).

#### 2.1.3 RNA Quantification and Qualification

Total RNA from each group was extracted using TRIzol Reagent (Sangon Biotech, China) and quantified and assessed for quality using the Agilent 2100 Bioanalyzer (Agilent Technologies, CA, USA).

#### 2.1.4 Library Preparation for Transcriptome Sequencing

Total RNA was then used as input material for RNA sample preparation. Briefly, mRNA was purified from total RNA using poly‐T oligo‐attached magnetic beads. Fragmentation was carried out using divalent cations under elevated temperature in the First Strand Synthesis Reaction Buffer (5×). First‐strand cDNA was then generated with a random hexamer primer and M‐MuLV Reverse Transcriptase, followed by RNA degradation using RNase H. Second‐strand cDNA synthesis was performed using DNA Polymerase I and dNTPs. Any remaining overhangs were converted into blunt ends through exonuclease/polymerase activities. After the adenylation of the 3′ ends of DNA fragments, adaptors with hairpin loop structures were ligated to prepare for hybridization. To select cDNA fragments with lengths ranging from 370 to 420 bp, the library fragments were purified using the AMPure XP system (Beckman Coulter, Beverly, USA). After PCR amplification, the PCR product was purified using AMPure XP beads, and the library was obtained.

#### 2.1.5 Clustering and Sequencing

After the library was qualified, the different libraries were pooled according to their effective concentrations and the target data output. The pooled libraries were then sequenced using the Illumina NovaSeq 6000, generating 150 bp paired‐end reads.

### 2.2 Data Downloading and the DFRGs Identification

Two RA datasets (GSE77298 and GSE55457) were downloaded from the Gene Expression Omnibus (GEO) database (https://www.ncbi.nlm.nih.gov/geo/) for analysis [11]. We noted biological heterogeneity in sample type and technical heterogeneity in detection platforms between the local PBMC RNA‐seq data used for model construction and the public GEO datasets used for independent validation. GSE77298 contains 16 RA samples and 7 healthy control samples based on the Affymetrix Human Genome U133 Plus 2.0 Array platform. GSE55457 contains 13 RA samples and 10 healthy control samples based on the Affymetrix Human Genome U133A Array platform, with samples derived from synovialtissue. To address this heterogeneity, internally standardized gene expression values were used within each dataset for comparison, and we focused on gene signals showing consistent patterns across PBMCs in the discovery set and synovial tissue in the validation datasets, thereby enhancing the robustness and generalizability of the study findings.

To further evaluate the cross‐tissue applicability of our findings, we included a third dataset GSE68689, which contains whole blood samples from 16 RA patients and 5 healthy controls profiled on the GPL20171 platform. DAS28 scores are available for the RA patients in this dataset. GSE68689 was used to validate the expression patterns of key DFRGs and the diagnostic performance of the constructed model, as whole blood and PBMCs share immune‐cell‐derived transcriptomic signals, particularly for immune‐related genes.

Ferroptosis‐related genes (FRGs) were obtained from the FerrDb website (http://www.zhounan.org/ferrdb/) [12], and disulfidptosis‐related genes (DRGs) were collected from a previous study [7]. Spearman correlation coefficients between the expression levels of DRGs and FRGs were calculated using the “cor.test()” function in R [13], with the threshold set to “|cor| > 0.3 and *p* < 0.05”. A total of 201 DFRGs were identified. Differentially expressed DFRGs were identified using the “limma” R package (version 3.40.6) with the threshold “|log_2_FC| > 0.5 and *p* < 0.05” [14]. The results were visualized as a volcano plot using the “ggplot2” R package.

### 2.3 Functional Enrichment Analysis

For enrichment analysis of differential DFRGs, we utilized the KEGG REST API (https://www.kegg.jp/kegg/rest/keggapi.html) to obtain the latest KEGG pathway gene annotations as the gene mapping background. The R package clusterProfiler (version 3.14.3) was employed to perform enrichment analysis, and gene set enrichment results were considered statistically significant at thresholds of *p* < 0.05 and false discovery rate (FDR) < 0.1 [14]. For functional enrichment analysis of DFRGs, GO annotations for DFRGs were retrieved from the org.Hs.eg.db R package (version 3.1.0). Genes were mapped to the background set, followed by enrichment analysis using the clusterProfiler R package. Similarly, statistical significance was defined as *p* < 0.05 and FDR < 0.05.

### 2.4 Infiltration Analysis of Immune Cells and Functions

To comprehensively characterize the immune microenvironment in RA, we performed systematic immune infiltration analyses. The single‐sample Gene Set Enrichment Analysis (ssGSEA) algorithm was employed to estimate the relative abundance of immune cell populations and the activity of immune‐related functions [15]. This method is particularly suited for analyzing individual samples, as it calculates an enrichment score by ranking genes within each sample based on their expression levels and comparing them against predefined gene sets representing specific immune cell types or functions. We calculated ssGSEA scores for 16 immune cell types and 13 immune‐related functions using well‐established and widely recognized immune cell marker gene sets. To visualize the overall patterns of immune infiltration, a heatmap was generated using the “pheatmap” R package [16]. Box plots, created with the “ggpubr” R package [17], were used to compare and visualize the differences in ssGSEA scores for each immune feature between the healthy control and RA groups. Group differences were statistically evaluated using the Wilcoxon rank‐sum test. To account for multiple comparisons, *p*‐values were adjusted using the Benjamini–Hochberg FDR method, and an adjusted *p*‐value (FDR) < 0.05 was considered statistically significant.

To investigate the interactions between key genes and the immune microenvironment, Spearman rank correlation analysis was conducted between the expression levels of the genes of interest and the ssGSEA scores of various immune cells and immune‐related functions. The results of these correlations were visualized in a heatmap using the “corrplot” R package. Furthermore, to clearly depict the strength and direction of these associations, a lollipop chart was generated, and representative correlations were illustrated with scatter plots. *p*‐values from correlation analyses were also adjusted using the FDR method, with statistical significance defined as an adjusted *p*‐value < 0.05.

### 2.5 Construction of Diagnostic Model

To build a predictive model for RA diagnosis, we initially analyzed 11 differentially expressed DFRGs. Spearman rank correlation analysis (R package “ggcorrplot”) was first performed to evaluate their associations with immune cell infiltration and immune functions, with *p*‐values adjusted using the FDR method. Genes showing significant correlations (adjusted *p* < 0.05) with key immune microenvironment features were prioritized. Subsequently, a feature selection process was conducted using a logistic regression model (a generalized linear model) to assess the independent contribution of these genes to RA risk. To refine the model and identify the most stable feature set, a stepwise selection procedure based on the Akaike Information Criterion (AIC) was applied to the candidate genes from the correlation analysis. This process identified five key genes (DUOX1, DRD4, ATP6V1G2, ASNS, CDKN1A) for the final model. The results of the logistic regression model were incorporated into a nomogram (R package “rms”) to visually estimate an individual's probability of having RA. Model performance was evaluated using receiver operating characteristic (ROC) curve analysis (R package “pROC”), and the area under the curve (AUC) was calculated to quantify the model's diagnostic accuracy in distinguishing RA patients from healthy controls.

### 2.6 Validation of Diagnostic Model

The GSE68689 (whole blood) dataset, along with the GSE77298 and GSE55457 (synovial tissue) datasets, were used to validate the diagnostic model constructed based on the five feature genes.

### 2.7 Construction of miRNA‐mRNA Interaction Network

MiRNet (www.mirnet.ca) [18], an integrated platform of data from 11 different miRNA databases, was used to predict miRNAs regulating the common hub genes. The database was used to screen for miRNAs targeting the five feature genes.

### 2.8 Prediction of Drug‐Gene Interaction and Identification of Potential Drugs

Using the DGIdb database (https://www.dgidb.org/) [19], five feature genes were further selected as potential druggable targets for the treatment of RA.

### 2.9 Single‐Cell Mendelian Randomization (scMR) Analysis

To explore the potential causal association between the expression levels of feature genes in specific immune cell subpopulations and the risk of RA, this study employs a two‐sample Mendelian Randomization (MR) analysis framework. This method leverages heritable variation strongly associated with gene expression as an instrumental variable to infer the causal effect of exposure (gene expression) on the outcome (RA) [20]. The selection of instrumental variables adheres to the following selection criteria: First, cis‐expression quantitative trait locus (cis‐eQTL) data were obtained from the OneK1K single‐cell eQTL database (https://onek1k.org/), which contains single‐cell transcriptomic and genetic data from peripheral blood mononuclear cells and provides cell‐type‐specific eQTL information for major immune cell subsets [21]. This resource enables the identification of genetic variants associated with gene expression in specific immune cell populations, making it suitable for evaluating immune‐cell‐specific regulatory effects in RA.

In this study, cis‐expression quantitative trait loci (cis‐eQTLs) associated with the expression of feature genes in relevant immune cell subpopulations were used as instrumental variables. We selected conditionally independent cis‐eQTLs significantly associated with their corresponding eGenes (*p* < 0.05). To ensure the independence of instrumental variables and mitigate bias arising from linkage disequilibrium (LD), we performed LD clustering analysis on the selected single nucleotide polymorphisms (SNPs). We removed highly correlated SNPs using a clustering *r* ^2^ threshold set at 0.01. The 1000 Genomes Project European population (EUR) was used as the LD reference panel for the following reasons: the RA GWAS summary statistics used in this study are derived from the FinnGen project, where the study participants are predominantly of European ancestry. Given the substantial differences in LD structure across ancestral populations, using an ancestry‐matched LD reference panel allows for a more accurate assessment of SNP correlations, optimizes the LD clumping process, and reduces potential bias in instrumental variable selection. Moreover, the OneK1K single‐cell eQTL data used in this study are also predominantly from European‐ancestry individuals. Therefore, using the EUR reference panel ensures ancestral consistency among the exposure data, outcome data, and LD reference data, thereby enhancing the reliability of the MR analysis results. Finally, the F‐statistic was used to evaluate the predictive strength of each retained instrumental variable. According to widely accepted standards, instrumental variables with an F‐statistic below 10 are considered to have limited power and may be subject to weak instrument biasand were therefore excluded from subsequent MR analyses. This series of rigorous selection processes aims to ensure the validity and reliability of the selected instrumental variables, laying a solid methodological foundation for subsequent causal inference.

### 2.10 Statistical Analysis

All statistical analyses in this study were performed using the R software environment (version 4.2.2), with the specific R packages detailed in Table S2. To evaluate the associations between DRGs and FRGs, as well as between differentially expressed DFRGs and immune cell infiltration and function, Spearman rank correlation analysis was employed. The resulting *p*‐values were adjusted for multiple comparisons using the FDR method, where statistical significance was defined as an adjusted *p*‐value < 0.05. In the MR analysis, the inverse variance weighting (IVW) method was primarily used to estimate causal effects, which provides unbiased estimates under the assumption that all instrumental variables are valid. Effect sizes are reported as odds ratios (OR) with corresponding 95% confidence intervals (CI). Additionally, to assess the robustness of the results and test for potential pleiotropy bias, we performed sensitivity analyses using the weighted median method and MR‐Egger regression. In the statistical analysis, a *p*‐value < 0.05 was considered statistically significant. The public databases used in this study are listed in Table S3.

## 3 Results

### 3.1 Differential DFRGs Between RA Patients and Healthy Controls

**FIGURE 2:**
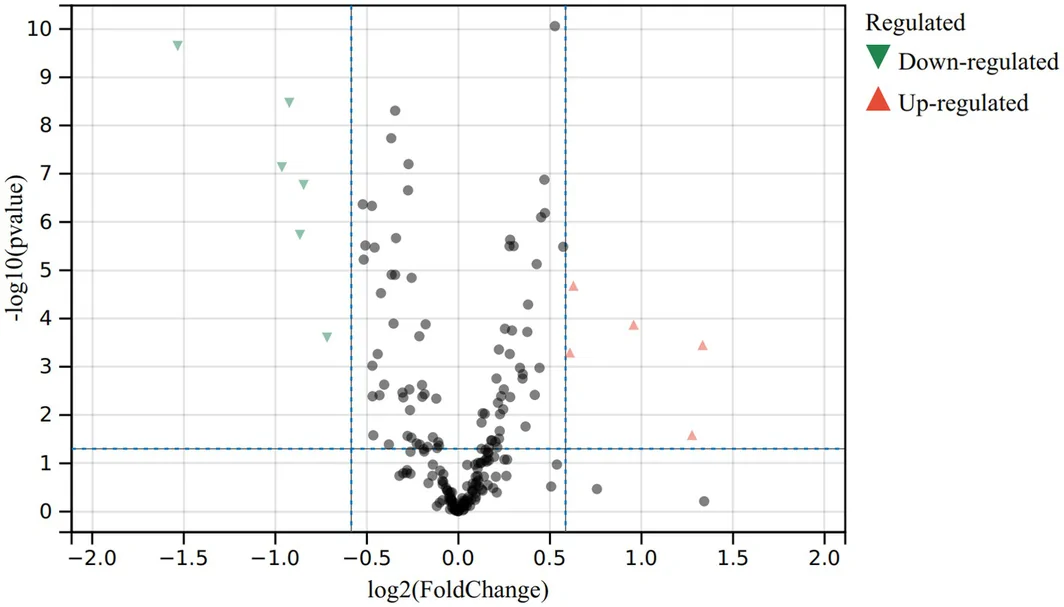
Identification of differentially expressed DFRGs between RA and HC groups. (A) Volcano plot showing differentially expressed DFRGs. DFRGs, disulfidptosis‐ and ferroptosis‐related genes; HC, healthy controls; RA, rheumatoid arthritis.

RNA sequencing data were obtained for 154 RA patients and 43 healthy controls. A total of 259 FRGs were identified by removing duplicate genes in three subgroups (driver, repressor, and signature) from the FerrDb website. DRGs were obtained from a previous study [7]. Spearman correlation analysis identified 201 DFRGs using the “cor.test()” function in R. Based on the criteria |log_2_FC| > 0.5 and *p* < 0.05, 11 differential DFRGs (HAMP, DUOX1, DRD4, ATP6V1G2, ASNS, CDKN1A, CAV1, ANGPTL7, HBA1, RRM2, PRKAA2) were identified, of which five (PRKAA2, HBA1, CAV1, CDKN1A, RRM2) were upregulated and six (HAMP, DUOX1, DRD4, ATP6V1G2, ASNS, ANGPTL7) were downregulated. The results are visualized in the volcano plot (Figure 2).

### 3.2 Functional Analysis of the Differential DFRGs

**FIGURE 3:**
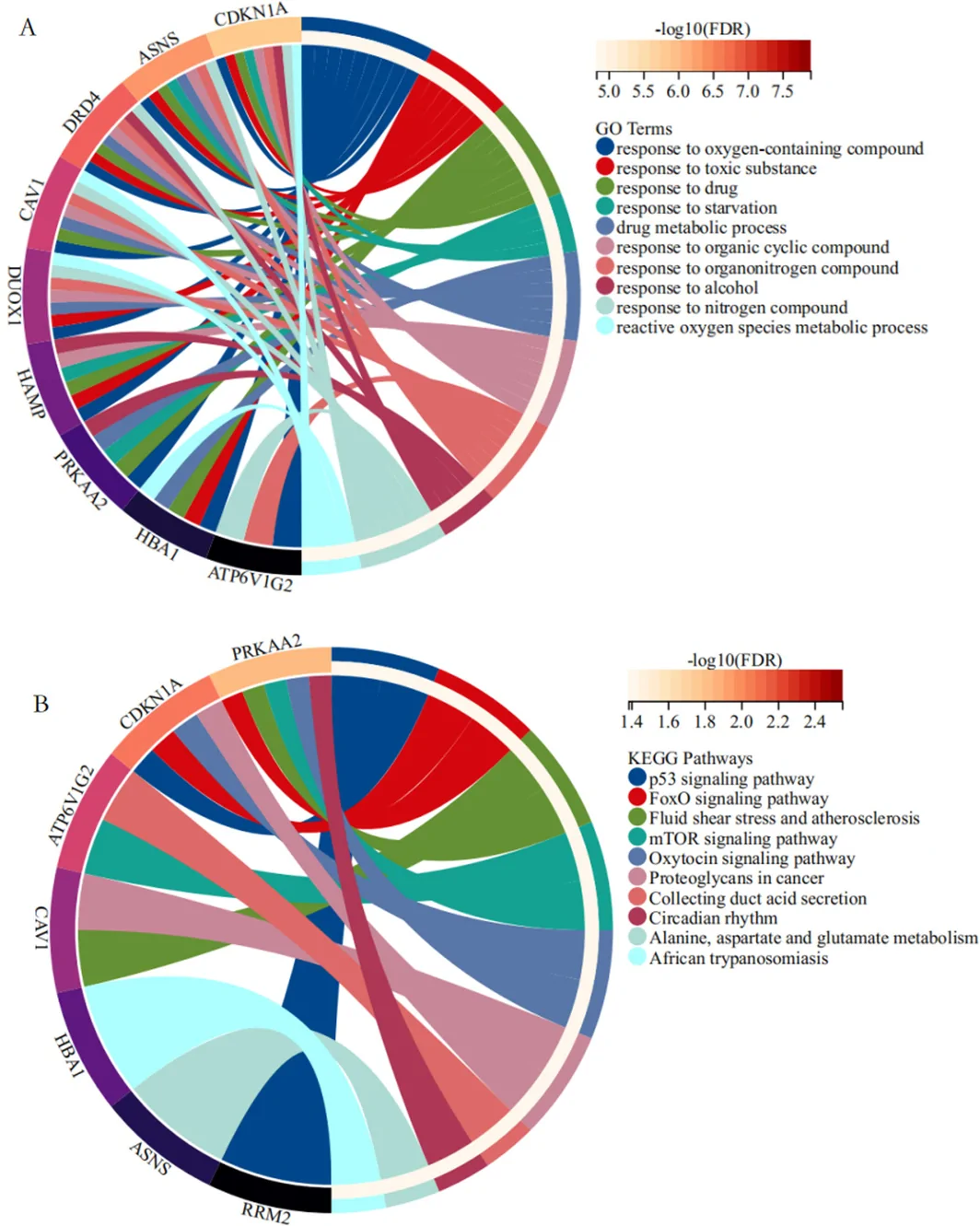
Functional enrichment analysis of differentially expressed DFRGs. (A) Gene Ontology (GO) enrichment analysis of differentially expressed DFRGs. (B) Kyoto Encyclopedia of Genes and Genomes (KEGG) pathway enrichment analysis of differentially expressed DFRGs. The outer gene labels represent enriched DFRGs, and colored ribbons indicate the associations between genes and corresponding GO terms or KEGG pathways. The color gradient indicates enrichment significance based on −log_10_(FDR). DFRGs, disulfidptosis‐ and ferroptosis‐related genes.

Functional enrichment analysis indicated that the differential DFRGs were primarily involved in the response to oxygen‐containing compounds, toxic substances, drugs, drug metabolism, organic cyclic compounds, organonitrogen compounds, alcohol, and nitrogen compounds (GO analysis threshold: FDR < 0.05, Figure 3A). In addition, the KEGG pathway analysis revealed that the differential DFRGs were enriched in the p53 signaling pathway, FoxO signaling pathway, fluid shear stress and atherosclerosis, mTOR signaling pathway, oxytocin signaling pathway, proteoglycans in cancer, collecting duct acid secretion, circadian rhythm, alanine, aspartate, and glutamate metabolism, and African trypanosomiasis (KEGG analysis threshold: FDR < 0.1, Figure 3B).

### 3.3 Infiltration Analysis of Immune Cells and Functions

**FIGURE 4:**
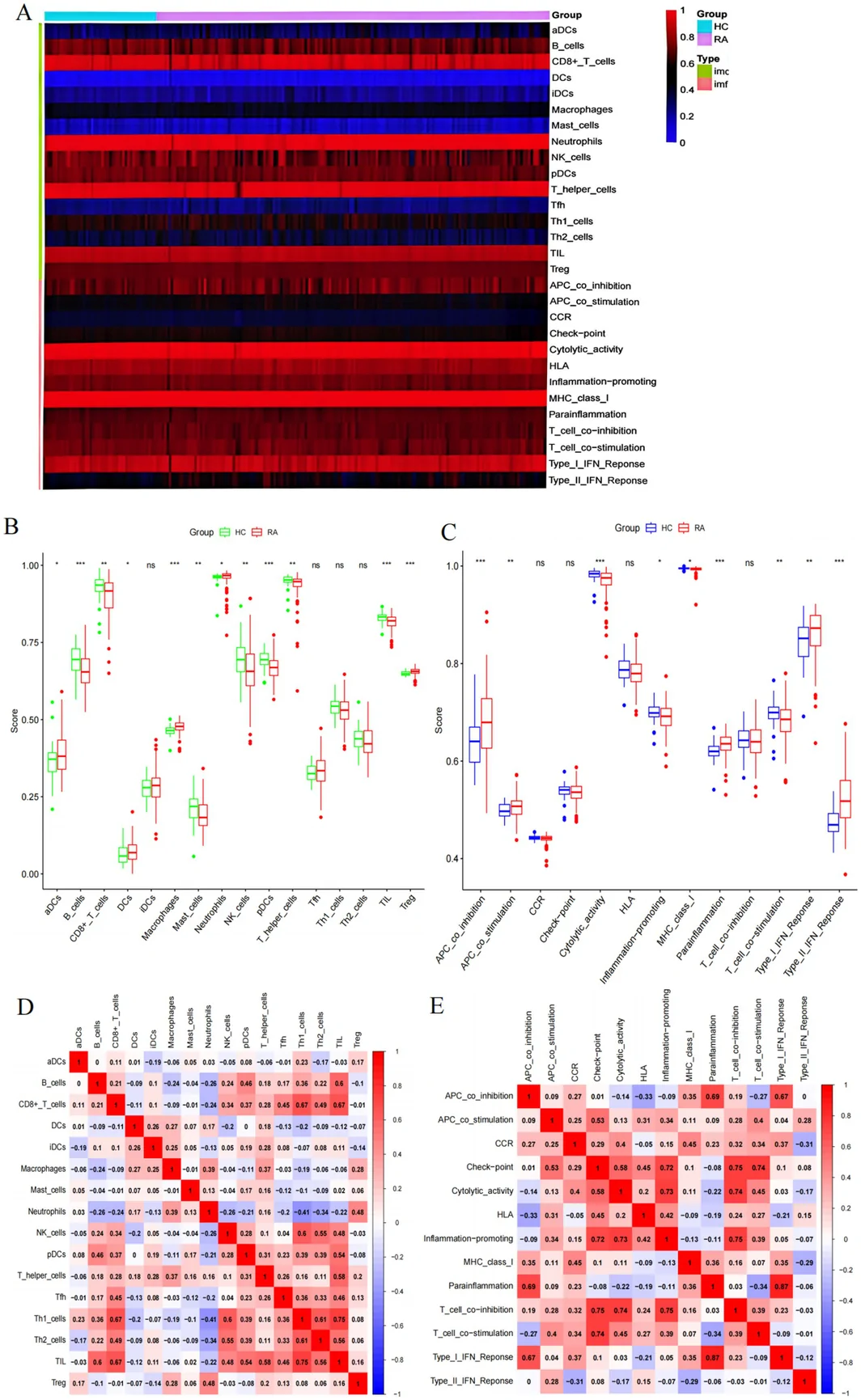
Differential immune cell infiltration and immune function analysis between RA patients and healthy controls. (A) Heatmap of differentially infiltrated immune cells and immune functions. (B) ssGSEA scores of 16 immune cell types. (C) ssGSEA scores of 13 immune‐related functions. (D) Correlation matrix of 16 immune cell types. (E) Correlation matrix of 13 immune‐related functions. *p < 0.05, **p < 0.01, ***p < 0.001. aDCs, activated dendritic cells; APCs, antigen‐presenting cells; CCR, CC chemokine receptor; DCs, dendritic cells; HLA, human leukocyte antigen; iDCs, immature dendritic cells; MHC, major histocompatibility complex; NK cells, natural killer cells; pDCs, plasmacytoid dendritic cells; ssGSEA, single‐sample Gene Set Enrichment Analysis; Tfh, follicular helper T cells; TILs, tumor‐infiltrating lymphocytes; Tregs, regulatory T cells.

To investigate differences in the immune microenvironment between RA patients and healthy controls, we conducted ssGSEA‐based immune infiltration analysis. The results revealed significant differences in immune cell infiltration and immune‐related functional activities between the two groups (Figure 4A–C). After FDR correction, 10 of the 16 immune cell populations showed significant differences. Specifically, macrophages and regulatory T cells (Tregs) were significantly increased in RA patients, whereas B cells, CD8+ T cells, mast cells, neutrophils, NK cells, plasmacytoid dendritic cells (pDCs), T helper cells, and tumor‐infiltrating lymphocytes (TILs) were significantly decreased (FDR < 0.05). Detailed statistical results, including mean values, median values, direction of change, raw *p*‐values, and FDR‐adjusted *p*‐values, are provided in Table S4.

Among the 13 immune‐related functional signatures, nine showed significant differences between RA patients and healthy controls after FDR correction. APC co‐inhibition, APC co‐stimulation, parainflammation, Type I IFN response, and Type II IFN response were significantly increased in RA patients, whereas cytolytic activity, inflammation‐promoting activity, MHC class I, and T‐cell co‐stimulation were significantly decreased (FDR < 0.05). Detailed statistical results are summarized in Table S5.

**FIGURE 5:**
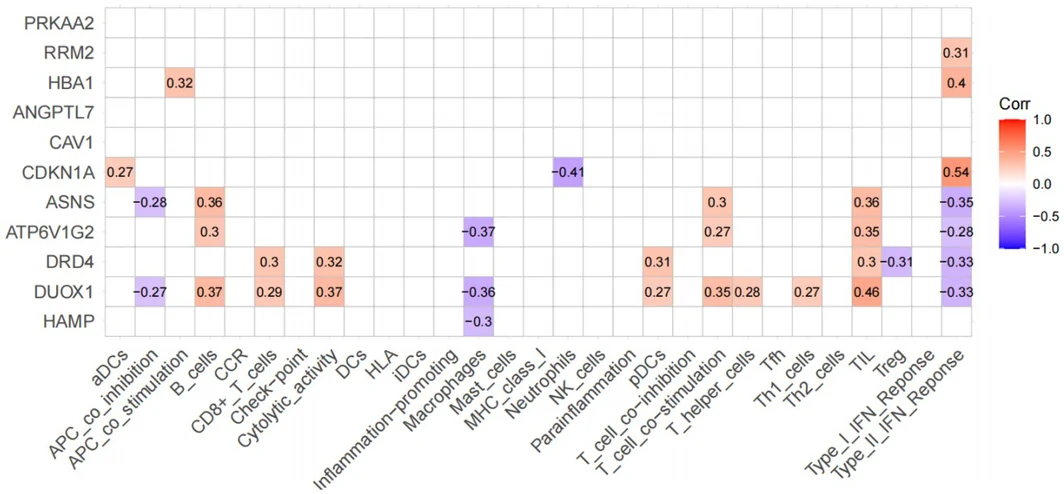
Correlation between hub DFRGs and immune cell infiltration or immune functions. Heatmap showing correlations among 11 hub DFRGs and immune‐related features. aDCs, activated dendritic cells; APCs, antigen‐presenting cells; CCR, CC chemokine receptor; DCs, dendritic cells; HLA, human leukocyte antigen; iDCs, immature dendritic cells; MHC, major histocompatibility complex; NK cells, natural killer cells; pDCs, plasmacytoid dendritic cells; Tfh, follicular helper T cells; TILs, tumor‐infiltrating lymphocytes; Tregs, regulatory T cells.

Further Spearman correlation analysis with FDR correction was performed to assess the associations between feature gene expression and immune infiltration scores. Only statistically significant correlations with adjusted *p* < 0.05 were displayed in the correlation heatmap (Figure 5). Notably, Type II IFN response showed a significant positive correlation with CDKN1A expression, while DUOX1 expression was significantly associated with TIL infiltration. These findings suggest that the identified feature genes may be closely associated with immune microenvironment remodeling in RA.

### 3.4 Identification of Five Feature Genes for Diagnosing RA and Validation of Diagnostic Model

**FIGURE 6:**
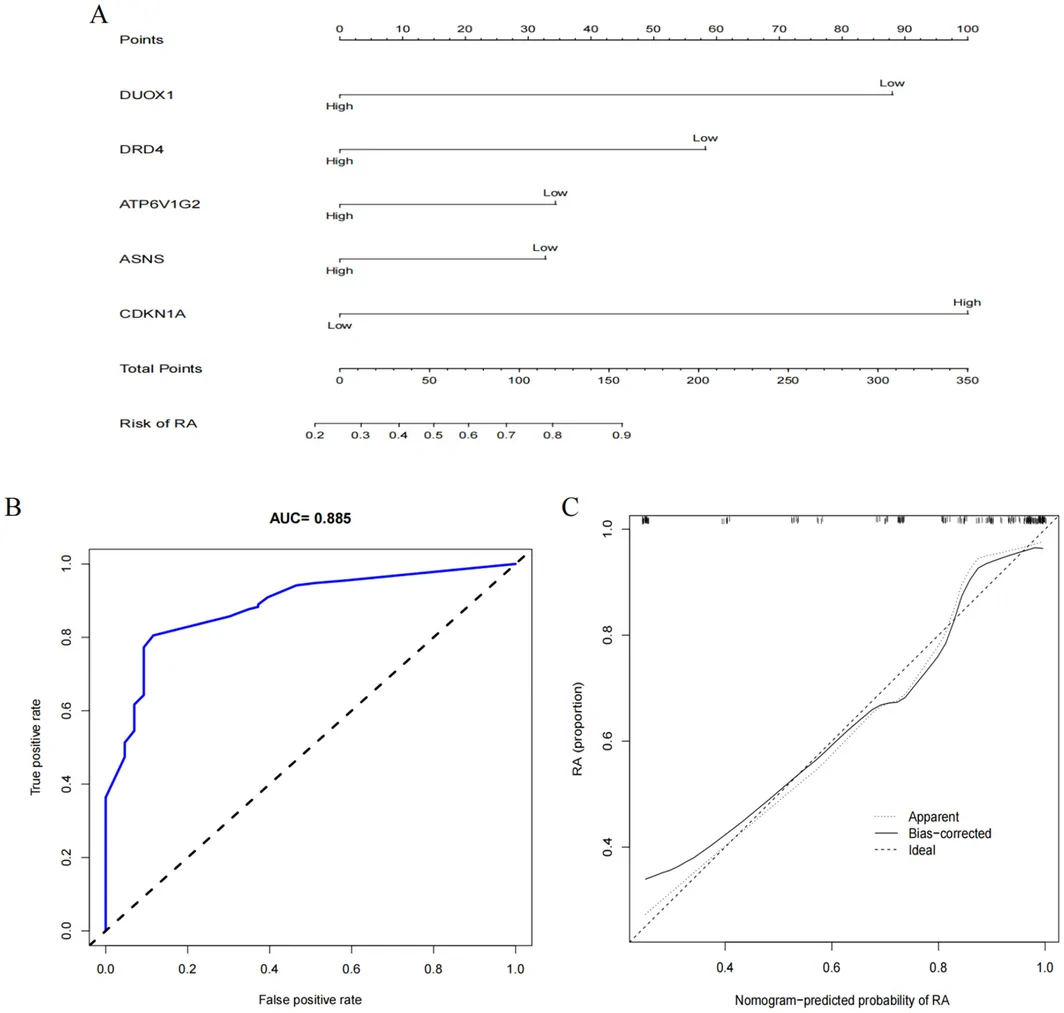
Construction of the diagnostic model. (A) Nomogram constructed based on five key feature genes. (B) Receiver operating characteristic (ROC) curve evaluating the diagnostic performance of the model. (C) Calibration curve assessing the predictive accuracy of the model. ROC, receiver operating characteristic.

To clarify the feature gene selection process and enhance transparency, we detail our stepwise statistical filtering pipeline. Starting from the initial 11 differentially expressed genes, we first performed Spearman rank correlation analysis to evaluate their associations with immune cell infiltration and functional activities, with *p*‐values adjusted using the FDR method. Genes showing significant correlations (adjusted *p* < 0.05) with key immune microenvironment features were prioritized. We then assessed the independent contribution of these candidate genes to RA status using a logistic regression model. To optimize the model and identify the most stable feature set, a stepwise selection procedure based on the AIC was applied. This process culminated in the selection of five key genes (DUOX1, DRD4, ATP6V1G2, ASNS, and CDKN1A). A logistic regression model based on these five genes was constructed for RA diagnosis prediction and visualized as a nomogram (Figure 6A). The diagnostic performance of this five‐gene signature was evaluated using the ROC curve, yielding an AUC of 0.885 in the training dataset (Figure 6B). Calibration curve analysis further indicated acceptable agreement between the nomogram‐predicted probability and the observed RA status in the training cohort (Figure 6C).

**FIGURE 7:**
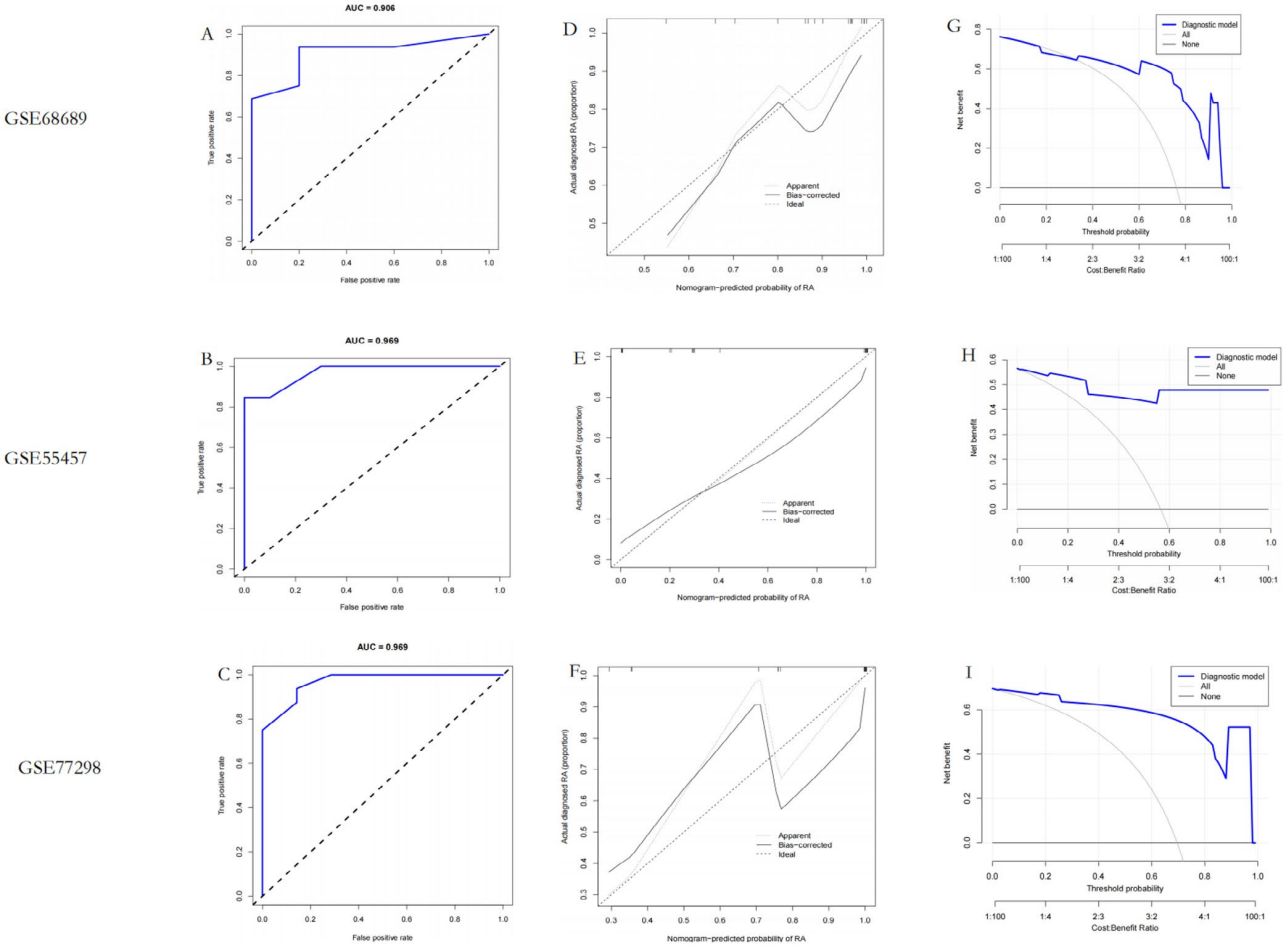
Performance evaluation of the Disulfidptosis‐ and Ferroptosis‐Related Gene signature for rheumatoid arthritis (RA) diagnosis. (A–C) Receiver operating characteristic (ROC) curves of the diagnostic model in the external validation cohorts GSE68689, GSE55457, and GSE77298, respectively. The corresponding area under the curve (AUC) values are shown in each panel. (D–F) Calibration curves of the nomogram in the GSE68689, GSE55457, and GSE77298 cohorts, respectively. The dotted line represents the apparent performance, the solid line indicates the bootstrap bias‐corrected performance, and the dashed diagonal line represents the ideal calibration. (G–I) Decision curve analysis (DCA) of the diagnostic nomogram in the three external validation cohorts. The blue line represents the diagnostic model, the gray line represents the strategy of treating all subjects as having RA, and the black horizontal line represents the strategy of treating no subjects as having RA. A higher net benefit indicates greater potential clinical utility across the corresponding threshold probabilities.

To further evaluate the robustness and generalizability of the proposed diagnostic model, three independent external cohorts, including GSE68689, GSE77298, and GSE55457, were used for validation. ROC analysis demonstrated consistently strong discriminatory performance across the validation datasets. The model achieved an AUC of 0.906 in GSE68689, while both GSE77298 and GSE55457 yielded an AUC of 0.969 (Figure 7A–C). Calibration curve analysis demonstrated satisfactory agreement between predicted probabilities and observed outcomes in all validation cohorts, indicating favorable calibration performance (Figure 7D–F). Furthermore, decision curve analysis (DCA) showed that the diagnostic model consistently provided greater net clinical benefit than the treat‐all and treat‐none strategies across a broad range of threshold probabilities (Figure 7G–I).

Taken together, these findings support the robustness, stability, and potential clinical applicability of the proposed five‐gene diagnostic model for RA. Nevertheless, further prospective multicenter studies are still warranted to validate the accuracy, reliability, and clinical utility of this model before clinical application.

### 3.5 Prediction of Potential miRNAs Targeting Feature Genes

**FIGURE 8:**
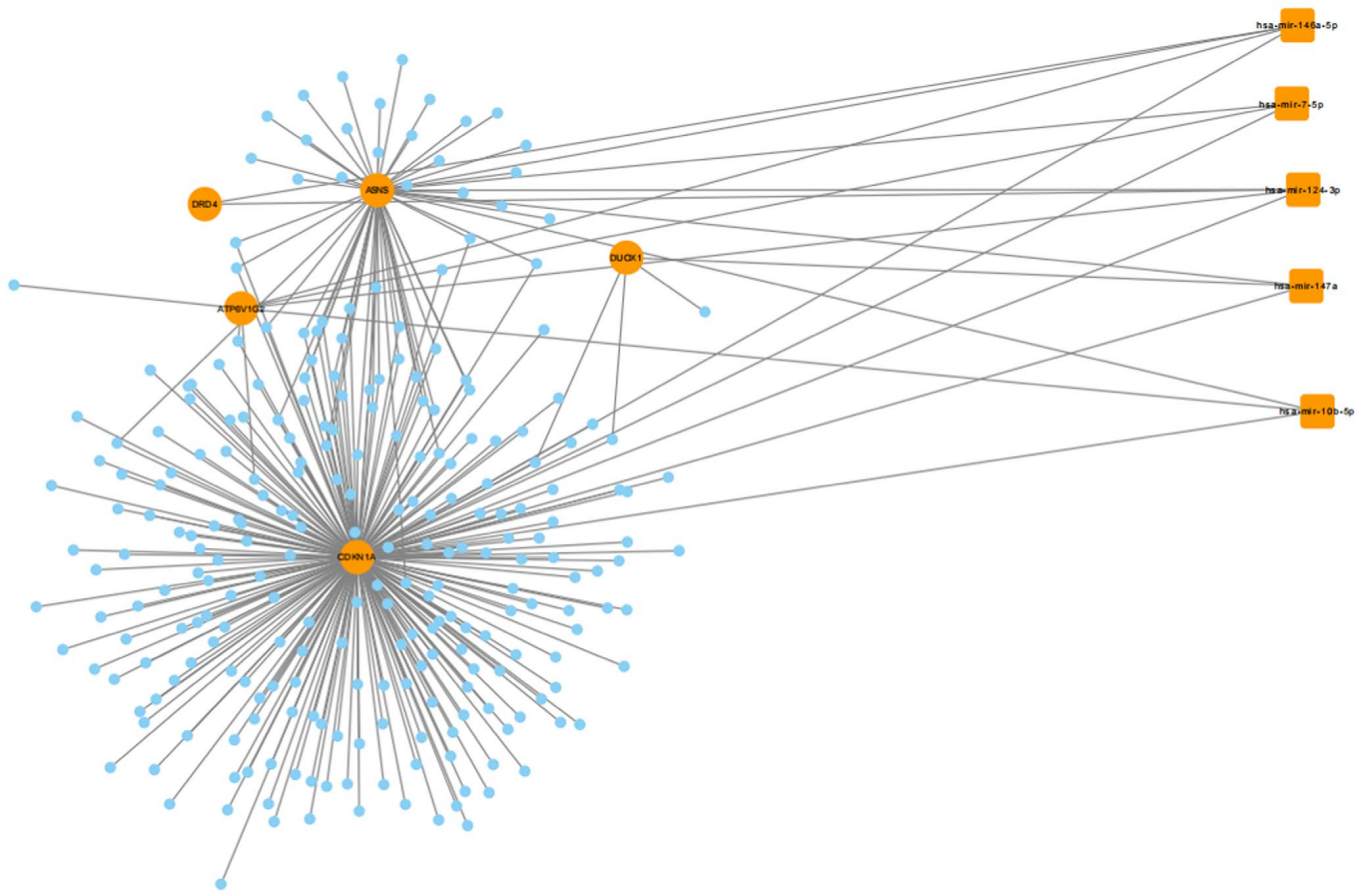
Prediction of potential miRNAs targeting feature genes. Interaction network between five feature genes and their predicted target miRNAs. Target miRNAs were identified using the miRNet database. miRNAs, microRNAs.

The miRNet database was used to screen miRNAs targeted by DUOX1, DRD4, ATP6V1G2, ASNS, and CDKN1A. As shown in Figure 7, a total of 304 miRNAs were predicted, five of which were associated with three or more genes (Figure 8). Specifically, CDKN1A interacts with 234 miRNAs, including hsa‐let‐7a‐5p, hsa‐miR‐16‐5p, and hsa‐miR‐17‐5p, among others. ASNS interacts with 57 miRNAs, including hsa‐miR‐17‐5p and hsa‐miR‐23a‐3p. ATP6V1G2 can be regulated by all seven miRNAs: hsa‐miR‐27a‐3p, hsa‐miR‐27b‐3p, hsa‐miR‐361‐5p, hsa‐miR‐10b‐5p, hsa‐miR‐124‐3p, hsa‐miR‐146a‐5p, and hsa‐miR‐7‐5p. DUOX1 has been shown to interact with four miRNAs: hsa‐miR‐148b‐3p, hsa‐miR‐152‐3p, hsa‐miR‐760, and hsa‐miR‐147a. DRD4 interacts with two miRNAs: hsa‐miR‐124‐3p and hsa‐miR‐146a‐5p.

### 3.6 Prediction of Drug‐Gene Interaction and Identification of Potential Drugs

**FIGURE 9:**
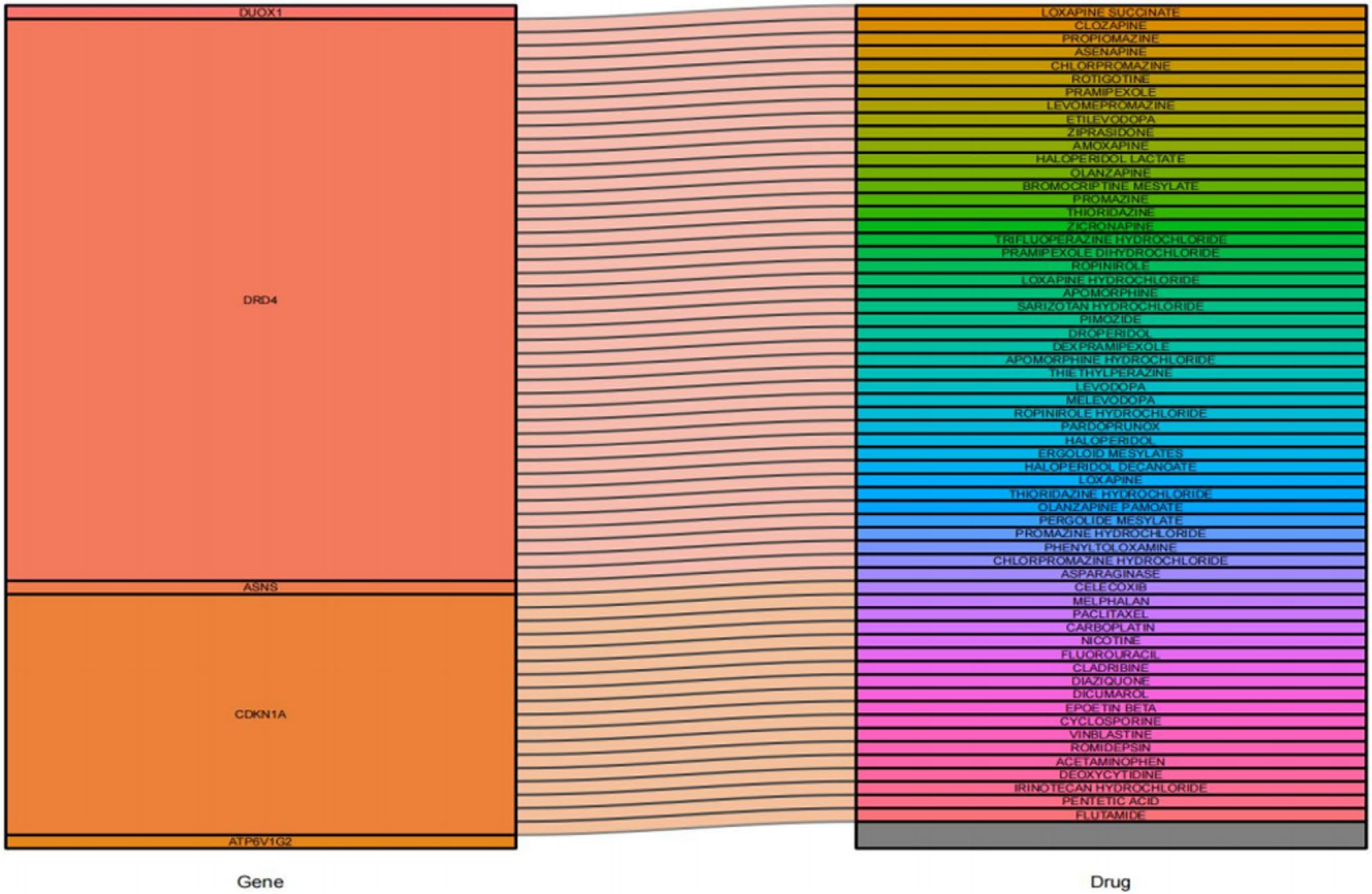
Drug–gene interaction network and prediction of potential therapeutic agents. Drug–gene interaction analysis of five feature genes. Potential target drugs or compounds were identified using the DGIdb database.

Five feature genes were selected as potentially targetable genes for RA therapy. The drug‐gene interaction results obtained through the DGIdb database revealed 61 potentially targeted drugs/compounds for RA treatment. Figure 8 displays these 61 drugs/compounds, ranked according to the “interaction group score” in the DGIdb database. Among them, 42 drugs targeted DRD4, with one having the highest prediction score. Eighteen drugs targeted CDKN1A, and one drug targeted ASNS. No potential drugs were identified for DUOX1 and ATP6V1G2 (Figure 9).

### 3.7 Causal Effect Analysis of Core Genes on the Risk of RA

**FIGURE 10:**
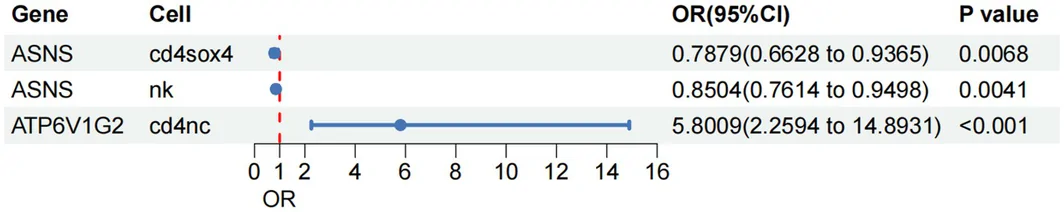
Forest plot illustrating the association between selected gene expression levels in specific immune cell subsets and disease risk. Odds ratios (ORs) with corresponding 95% confidence intervals (CIs) are presented for each gene–cell pair. Points represent estimated ORs, and horizontal bars indicate 95% CIs. Statistical significance was assessed using logistic regression analysis, with corresponding *p*‐values shown on the right. Genes with OR > 1 indicate increased risk, whereas OR < 1 suggests a protective effect.

To explore the potential causal impact of core gene expression on the risk of RA at the genetic level, we conducted a two‐sample MR analysis based on public sc‐eQTL data. The analysis results showed that the expression levels of core genes in specific immune cell subpopulations exert significant and opposing causal effects on the risk of RA (Table 1). High expression of the ASNS gene in CD4+ SOX4+ T cells is protective against RA (OR = 0.79, 95% CI: 0.66–0.94, *p* = 0.007). Similarly, high expression of ASNS (Figure 10) in natural killer (NK) cells also confers a protective effect (OR = 0.85, 95% CI: 0.76–0.95, *p* = 0.004). In contrast, high expression of the ATP6V1G2 gene in CD4+ naïve T cells is strongly associated with increased RA risk, exhibiting a notably large effect size (OR = 5.80, 95% CI: 2.26–14.89, *p* = 2.58 × 10^−4^).

**TABLE 1:** Results of single‐cell Mendelian randomization (MR) analysis of core genes.

Gene	Cell	Nsnp	Method	B	Se	Or	Or_lci95	Or_uci95	*p*
ASNS	cd4sox4	4	Inverse variance weighted	−0.2384	0.0882	0.7879	0.6628	0.9365	0.0068
ASNS	nk	7	Inverse variance weighted	−0.1621	0.0564	0.8504	0.7614	0.9498	0.0041
ATP6V1G2	cd4nc	2	Inverse variance weighted	1.758	0.4811	5.8009	2.2594	14.8931	0.0003

Abbreviations: B, beta coefficient; Ci, confidence interval; Nsnp, number of single‐nucleotide polymorphisms; Or, odds ratio; Se, standard error.

## 4 Discussion

RA is a systemic autoimmune disease characterized by chronic inflammation of the synovium, with joint destruction and functional loss severely affecting approximately 1% of the global population [22]. The etiology of this disease involves complex interactions between immune regulation abnormalities and various cell death pathways, directly impacting patient prognosis and quality of life, and often accompanied by multiple comorbidities [23]. Despite advancements in existing treatment methods, effective biomarkers for predicting disease progression remain scarce [24]. In recent years, novel modes of cell death such as disulfidptosis and ferroptosis have emerged as topics of interest due to their potential roles in regulating redox homeostasis and the cytoskeleton, which are hypothesized to play important roles in the pathology of RA. Therefore, exploring the potential synergistic effects of these pathways in RA may help reveal new mechanisms of the disease and provide new directions for identifying diagnostic biomarkers and developing targeted therapies.

Notably, this study innovatively integrates DFRGs to analyze the molecular landscape of RA. We first identified 11 differentially expressed DFRGs in peripheral blood mononuclear cells of RA patients and constructed a novel diagnostic model based on five core genes (CDKN1A, DUOX1, DRD4, ATP6V1G2, ASNS). This model demonstrated excellent diagnostic performance across multiple independent validation cohorts from both whole blood (GSE68689, AUC = 0.906) and synovial tissue (GSE77298 and GSE55457, AUC = 0.969), overcoming the limitations of traditional serum biomarkers (such as RF and ACPA) through the integration of multi‐pathway information. Furthermore, this study integrates multiple orthogonal methods, including differential expression analysis, single‐cell transcriptome analysis, independent validation datasets, immune cell‐specific characterization, and MR analysis. MR analysis, in particular, leverages germline genetic variants fixed at conception, which are less susceptible to traditional confounders such as age. The consistency of results across these independent analytical platforms provides computational support for the reliability of our findings, although further experimental validation is needed to confirm these hypothesis‐driven observations.

These core genes form a central network that suggests the underlying mechanisms that may drive the synergistic regulation of RA “dual death” (ferroptosis and disulfidptosis). Specifically, CDKN1A, as a regulatory hub, may sensitize cells to disulfidptosis by depleting glutathione (GSH) while conferring resistance to ferroptosis, reflecting opposing regulation of the two death pathways [25]. DUOX1, as a primary source of oxidative stress, generates elevated levels of ROS that can directly trigger lipid peroxidation (ferroptosis) and induce disulfide bond accumulation (disulfidptosis), serving as an upstream switch for both pathways [26]. Meanwhile, ATP6V1G2 disrupts lysosomal acidification, simultaneously impairing the metabolism of ferroptosis‐related substrates and the clearance of disulfidptosis‐associated protein aggregates, thereby creating a permissive condition for both processes [27]. This synergistic interplay may be mediated by a coordinated mechanism in which DUOX1‐derived ROS and CDKN1A‐mediated GSH depletion may jointly promote disulfidptosis, while lysosomal dysfunction driven by ATP6V1G2 may amplify ferroptotic damage. Together, these processes may establish a self‐reinforcing loop in which DRD4 and ASNS may additionally serve as indirect modulators of immune cell sensitivity.

Consequently, these findings suggest that the identified genes may be associated with immune microenvironment remodeling in RA. Collectively, they may contribute toa shift from immune homeostasis to a pro‐inflammatory, self‐sustaining state [28, 29]: (1) Potential dysregulation of T‐cell balance: CDKN1A may be associated with enhanced type II interferon responses, which could contribute to disturbances in the Treg/Th17 equilibrium and favor a pathogenic Th1/Th17‐dominant immune state [25, 30]. (2) Potential innate immune activation and autoantibody production: DUOX1 has been reported to be associated with neutrophil NETosis and the release of citrullinated antigens, which may facilitate B‐cell activation and ACPA production [26, 31]. (3) Potential impairment of immune tolerance and phagocytic function: ATP6V1G2 may influence lysosomal acidification, which has been reported to be associated with pro‐inflammatory M1 macrophage polarization and impaired Treg function, thereby potentially contributing to the disruption of immune homeostasis in RA [32].

To complement our observational findings and explore potential causal relationships at the cellular level, we conducted scMR analysis. This method utilizes genetic variants as instrumental variables to infer potential causal effects of the exposure on the outcome within specific cell types, offering a supplementary line of evidence from association studies. The scMR analysis suggested potential causal roles for some genes in specific immune cell contexts; for instance, genetic evidence supported ASNS as a potential protective factor in CD4+ SOX4+ T cells and NK cells, while ATP6V1G2 was indicated as a potential risk factor in CD4+ naïve T cells. We acknowledge that the analysis for some gene‐cell pairs had limited statistical power. The observed downregulation of ASNS in RA tissues coupled with its inferred protective role in certain cells highlights the complexity of its function, which may be cell type‐specific or influenced by post‐transcriptional regulation. We cautiously speculate that in specific immune subpopulations, ASNS might influence metabolic pathways, whereas ATP6V1G2 may modulate immune cell activation. Overall, the application of scMR represents a methodological approach to move beyond bulk tissue signals and generate hypotheses regarding gene functions within discrete immune niches; these hypotheses warrant further validation [33].

The current study has several limitations. First, although DFRGs associated with RA were identified through integrated transcriptomic analysis, single‐cell analysis, external validation, and MR, the findings remain primarily based on computational inference and should be interpreted as hypothesis‐generating. Experimental validation is required to confirm the biological roles of these genes and the predicted therapeutic candidates in RA. Second, the age difference between HC and RA groups, the relatively relaxed differential expression threshold (|log_2_FC| > 0.5), and the exploratory nature of KEGG enrichment under a less stringent cutoff may influence result interpretation. Nevertheless, the final feature genes were further supported by correlation analysis, external validation datasets, and MR analysis, which strengthens the robustness of the identified candidate biomarkers. Future studies with larger age‐matched cohorts, functional experiments, and prospective clinical validation are warranted.

## 5 Conclusion

This study suggests that disulfidptosis‐ and ferroptosis‐related genes may mediate synergistic mechanisms in RA and develops a potentially highly accurate diagnostic model based on five key genes, which may lay the foundation for precise RA diagnosis, risk stratification, and targeted therapy. It reveals a possible pathogenic cycle linking oxidative stress, cell death, and immune microenvironment dysregulation, and identifies potential therapeutic targets including CDKN1A, DUOX1, and ATP6V1G2, providing genetic evidence that these genes may influence RA risk within specific immune cell subsets. However, these findings require further experimental validation.

## Funding

This work was supported by the National Natural Science Foundation of China (Grant No. 81771738), the Chongqing Talents Program (cstc2022ycjh‐bgzxm0139), the Program for Youth Innovation in Future Medicine of Chongqing Medical University (W0197), the Kuanren Talents Program of the Second Affiliated Hospital of Chongqing Medical University (13‐002‐017), the Sichuan Province Medical Scientific Research Project Plan (S23001), the Sichuan Medical Association Medical Research Project (S2024040), the Key Projects Fund of Science and Technology Department of Sichuan Province (2022YFS0250), the Health Commission of Sichuan Province Medical Science and Technology Program (24WSXT078), and the Sichuan Provincial Association of Integrative Medicine Research Project (No. ZXY2025039).

## Ethics Statement

This study followed the guidelines of the ethics committee and was approved by the Dazhou Central Hospital Ethics Committee (approval number: 2021‐022). All patients signed the informed consent.

## Conflicts of Interest

The authors declare no conflicts of interest.

## Supporting information


**Table S1:** The baseline characteristics between RA and HC.
**Table S2:** The R package used in the manuscript.
**Table S3:** Databases used in the manuscript.

**Table S4:** Statistical comparison of immune cell infiltration scores estimated by ssGSEA between rheumatoid arthritis (RA) patients and healthy controls.

**Table S5:** Statistical comparison of immune‐related functional signatures estimated by ssGSEA between rheumatoid arthritis (RA) patients and healthy controls.

## Data Availability

The raw RNA‐seq data from this study has been publicly released in our previously published work and can be accessed via the following link: https://ngdc.cncb.ac.cn/search/specific?db=bioproject&q=PRJCA011639. Other data supporting the findings of this study are available from the corresponding author upon reasonable request. The RNA‐Seq data have been deposited in the Gene Expression Omnibus (GEO) database under accession numbers GSE77298 and GSE55457. The data can be accessed via the following links: [GSE77298] (https://www.ncbi.nlm.nih.gov/geo/query/acc.cgi?acc=GSE77298), [GSE55457] (https://www.ncbi.nlm.nih.gov/geo/query/acc.cgi?acc=GSE55457).
