# Supplementary material for: Disulfidptosis‐ and Ferroptosis‐Related Gene Signatures in Rheumatoid Arthritis: Association Analysis and Diagnostic Model Construction
Source: Kaohsiung J Med Sci. 2026 Sep 3:e70287. Online ahead of print. doi: 10.1002/kjm2.70287 (PMC13540386; doi:10.1002/kjm2.70287)
Supplement: Supplementary file 1 [file KJM2-9999-e70287-s003.docx]

Supplementary Table 1 The baseline characteristics between RA and HC

| Characteristics | RA(N=154) | HC(N=43) | Total(N=197) | pvalue |
| --- | --- | --- | --- | --- |
| Gender |  |  |  | 0.32 |
| Female | 125(81.17%) | 32(74.42%) | 157(79.70%) |  |
| Male | 29(18.83%) | 11(25.58%) | 40(20.30%) |  |
| Age(year) | 55.02±11.27 | 49.77±4.57 |  | <0.0001 |
| CRP (mg/L) | 20.05±28.11 |  |  |  |
| RF(U/ML) | 231.18±312.06 |  |  |  |
| Anti-CCP(RU/ml) | 289.83±119.43 |  |  |  |
| ESR (mm/h) | 38.65±22.72 |  |  |  |
| DAS28 score | 4.47±1.36 |  |  |  |

Except where indicated otherwise, values are mean ±SD. RA, Rheumatoid arthritis; HC, healthy controls; CRP, Creactive protein; RF, Rheumatoid Factor; Anti-CCP, anti-cyclic peptide containing citrulline; ESR, Erythrocyte Sedimentation Rate; DAS28 score,28 Joint disease activity score

Supplementary Table 2 The R package used in the manuscript

| Name | Version | Fuctions |
| --- | --- | --- |
| Limma | 3.40.6 | Analysis of difference |
| ggplot2 | 3.4.4 | Visualization of data |
| clusterProfiler | 3.14.3 | Gene function enrichment analysis |
| org.Hs.eg.db | 3.1.0 | Annotation of genes |
| gsva | 1.50.0 | Gene set analysis tools |
| pheatmap | 1.0.12 | Draw a heat map |
| ggpubr | 0.6.0 | Draw a box plot |
| corrplot | 0.92 | Used to visualize the correlation matrix |
| ggcorrplot | 0.1.4.1 | Visual correlation matrix |
| rms | 6.7-1 | Constructing regression models |
| ROCR | 1.0-11 | Draw ROC curve |
| TwoSampleMR | 0.5.6 | Analysis of two-sample Mendelian randomization（MR） |

Supplementary Table 3 Databases used in the manuscript

| Name | Web site | Fuctions |
| --- | --- | --- |
| GEO | https://www.ncbi.nlm.nih.gov/geo/ | Gene expression database |
| FerrDb | http:// www.zhounan.org/ferrdb/ | Database of ferroptosis regulators and diseases |
| KEGG | https://www.kegg.jp/kegg/rest/keggapi.html | Comprehensive information on biological molecules such as genes,proteins, metabolites and signaling pathways is provided |
| miRNet | https://www.mirnet.ca/ | Prediction and functional analysis of microRNA |
| DGIdb | https://www.dgidb.org/ | Information on the association of genes with known or potential drugs is provided |
| OneK1K | https://onek1k.org/ | Obtain cis-expression quantitative trait loci (cis-eQTL) data. |

GEO, Gene Expression Omnibus database; KEGG, Kyoto Encyclopedia of Genes and Genomes; miRNet, a miRNA-centric network visual analytics platform; DGIdb, Drug-Gene Interaction database,OneK1K ,One Thousand and One Genomes.
